# Modeling, Fabrication, and Testing of a 3D-Printed Coriolis Mass Flow Sensor

**DOI:** 10.3390/s23084062

**Published:** 2023-04-18

**Authors:** Mahdieh Yariesbouei, Remco G. P. Sanders, Remco J. Wiegerink, Joost C. Lötters

**Affiliations:** 1 Integrated Devices and Systems, University of Twente, 7500 AE Enschede, The Netherlands; 2 Bronkhorst High-Tech BV, 7261 AK Ruurlo, The Netherlands

**Keywords:** 3D-printed tube, Coriolis mass flow sensor, circular cross-section

## Abstract

This paper presents the modeling, fabrication, and testing of a 3D-printed Coriolis mass flow sensor. The sensor contains a free-standing tube with a circular cross-section printed using the LCD 3D-printing technique. The tube has a total length of 42 mm, an inner diameter of about 900 µm, and a wall thickness of approximately 230 µm. The outer surface of the tube is metalized using a Cu plating process, resulting in a low electrical resistance of 0.5 Ω. The tube is brought into vibration using an AC current in combination with a magnetic field from a permanent magnet. The displacement of the tube is detected using a laser Doppler vibrometer (LDV) that is part of a Polytec MSA-600 microsystem analyzer. The Coriolis mass flow sensor has been tested over a flow range of 0–150 g/h for water, 0–38 g/h for isopropyl alcohol (IPA), and 0–50 g/h for nitrogen. The maximum flow rates of water and IPA resulted in less than a 30 mbar pressure drop. The pressure drop at the maximum flow rate of nitrogen is 250 mbar.

## 1. Introduction

In the past several decades, several microfluidic mass flow sensors were developed based on the Coriolis effect [1]. The most important advantages of Coriolis mass flow sensing compared to other flow sensing principles are the independence of pressure, flow profile, and fluid properties [2]. Most micro-Coriolis mass flow sensors are fabricated based on silicon micromachining techniques, such as anisotropic wet [3,4,5] and dry etching [2,6,7], and surface channel technology [8,9,10,11,12]. However, the high fabrication costs of these silicon micromachined sensors make them unsuitable for applications that need a disposable sensor. Furthermore, these fabrication methods result in channels with a hexagonal, rectangular, or semicircular cross-sectional shape, whereas, to decrease the deformation of the tube due to the pressure of the medium and increase the flow range of the sensor, a circular cross-section with a large diameter is needed [13]. Pressure dependency of the cross-sectional shape affects the stiffness of the tube structure, which results in measurement errors when used in resonant sensors such as a Coriolis mass flow sensor or a density sensor [14]. To reduce fabrication costs and increase the tube diameter, devices were developed based on polymer photolithography [15] and 3D printing [16]. However, these technologies resulted in a rectangular cross-sectional tube shape. To obtain a circular cross-sectional shape, electroplated nickel [17] tubes and glass capillaries [18] can be considered. In [17], it was shown that the nickel-plated tubes can in principle be used in a Coriolis mass flow sensor, but the demonstrated performance needs much improvement. Glass capillaries have been used successfully for density and mass sensing. However, these capillaries can only be fabricated as straight tubes [19,20,21,22].

In 2020, the first 3D-printed Coriolis mass flow sensor was proposed by Pagani [16]. This design contained a tube with a rectangular cross-sectional shape with inner dimensions of 1 mm × 2 mm and a wall thickness of 500 µm. However, this sensor was only tested with air at only two flow rates, 0 g/h and 150 g/h, which means it was only tested by detecting the effect of the on/off switching of airflow. As a result, it is not clear whether the change in the output signal is due to the applied mass flow or due to the changing pressure inside the tube.

In this paper, we present the modeling, fabrication, and measurement results of a 3D-printed micro-Coriolis mass flow sensor. First, in Section 2, we explain the design and basic operating principle of the Coriolis mass flow sensor. Next, in Section 3, the fabrication sequence is described in detail. Finally, in Section 4, we discuss the results of mass flow measurements performed with different fluids, water, IPA, and nitrogen, and compare these with the mathematical model to confirm that the device indeed measures mass flow.

## 2. Modeling and Design of the Sensor

### 2.1. Basic Structure and Operating Principle

**Figure 1:**
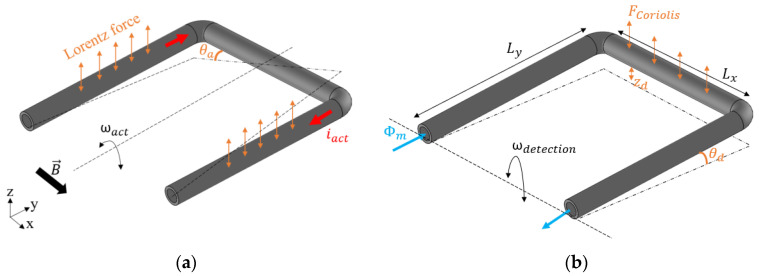
A U-shaped free-suspended tube that is fixed at both ends. (**a**) Twist mode vibration due to Lorentz force, and (**b**) swing mode vibration due to Coriolis force.$\;\omega_{act}$ and $\omega_{detection}$ are the actuation and detection mode of angular velocity, respectively.

The U-shaped tube structure and operation principle of the Coriolis mass flow sensor is shown in Figure 1. A U-shape was chosen because it has less corners in comparison to other common shapes used for Coriolis mass flow sensors, which makes it easier to print.

The sensor consists of a free-suspended tube that is fixed on both ends. To generate Lorentz forces and to bring the tube into vibration, an AC actuation current ($i_{act}$) is applied to the tube in the presence of a magnetic field (B). According to the direction of the magnetic field in this design, as shown in Figure 1a, the resulting Lorentz forces are in opposite directions at the left and right sides of the sensor tube. This will induce a twist mode vibration, indicated by the angular velocity $\omega_{act}$ and rotation angle $\theta_{a}$. The Lorentz force at each side of the tube can be expressed as:

(1) $$F_{act}=L_{y}\cdot \left|{\vec{i}}_{act}\times \vec{B}\right|=L_{y}B{\hat{i}}_{act}\cos\left(\omega_{a}t\right)\;$$

where $L_{y}$ is the length of the tube segments perpendicular to the magnetic field direction as indicated in Figure 1b, and ${\hat{i}}_{act}$ and $\omega_{a}$ are the amplitude and frequency of the actuation current. When driven in resonance, the angular displacement $\theta_{a}$ will have a 90 degrees phase shift with respect to the actuation current, and can be expressed as:

(2) $$\theta_{a}=\alpha \sin\left(\omega_{a}t\right)\;$$

where α is the amplitude of the actuation angle around the y-axis.

As shown in Figure 1b, when a medium is flowing through the vibrating tube, Coriolis forces will be generated and result in a secondary vibration of the tube in swing mode. The Coriolis force linearly depends on the mass flow rate,$\;\Phi_{m}$, and the amplitude of the resulting swing mode vibration will be proportional to mass flow.

The Coriolis force is given by:

(3) $${\vec{F}}_{coriolis}=-2L_{x}\left({\vec{\omega }}_{act}\times {\vec{\Phi }}_{m}\right)\;$$

where $L_{x}$ is the length of the tube segment which is perpendicular to the rotation axis. The actuation mode of angular velocity ${\vec{\omega }}_{act}$ is equal to $\frac{d{\vec{\theta }}_{a}}{dt}$.

The Coriolis force generates a torque along the x-axis. This detection torque is defined as:

(4) $$T_{d}=L_{y}\cdot F_{coriolis}$$

Taking the time derivative of angular displacement in actuation mode (2), inserting this in (3), and substituting in (4), we obtain:

(5) $$T_{d}=-2L_{x}L_{y}\Phi_{m}\omega_{a}\alpha \cos\left(\omega_{a}t\right)$$

This torque results in angular displacements $\theta_{d}$ in the detection mode, as indicated in Figure 1b. To obtain an estimate of the amplitude of the displacements in the detection mode, we can describe the detection mode by a simple lumped element second-order differential equation [23]:

(6) $$J_{d}\frac{d^{2}\theta_{d}\left(t\right)}{dt^{2}}+R_{d}\frac{d\theta_{d}\left(t\right)}{dt}+K_{d}\theta_{d}\left(t\right)=T_{d}\left(t\right)\;$$

where $J_{d}$ is the modal moment of inertia, $R_{d}$ is the modal damping coefficient, and $K_{d}$ is the modal spring constant, which can be expressed as:

(7) $$J_{d}=\frac{5}{9}mL_{y}^{2}$$

(8) $$R_{d}=\frac{K_{d}}{\omega_{d}Q_{d}}$$

(9) $$K_{d}=\omega_{d}^{2}J_{d}$$

with m as the total mass of the tube and the fluid inside it, and $Q_{d}$ and $\omega_{d}$ the quality factor and resonance frequency of the detection mode, respectively. The modal moment of inertia $J_{d}$ is calculated assuming that the tube is a slender rod because the diameter of the tube is much smaller than the length of the tube, and assuming that $L_{x}\approx L_{y}$.

Substituting (5) into (6), the solution for the detection angle $\theta_{d}$ will be in the form:

(10) $$\theta_{d}=A\sin\left(\omega_{a}t\right)+B\cos\left(\omega_{a}t\right)$$

Inserting (7) to (10) into (6) and solving for A and B results in:

(11) $$A=\frac{-2\;L_{x}L_{y}\Phi_{m}\omega_{d\;}\alpha \;Q_{d}\omega_{a}^{2}}{J_{d}\left[{\left(\omega_{d}^{2}-\omega_{a}^{2}\right)}^{2}Q_{d}^{2}+\omega_{a}^{2}\omega_{d}^{2}\right]}$$

(12) $$B=\frac{-2\;L_{x}L_{y}\Phi_{m}\omega_{a\;}\alpha \;Q_{d}^{2}\left(\omega_{d}^{2}-\omega_{a}^{2}\right)}{J_{d}\left[{\left(\omega_{d}^{2}-\omega_{a}^{2}\right)}^{2}Q_{d}^{2}+\omega_{a}^{2}\omega_{d}^{2}\right]}$$

Substituting (11) and (12) into (10), the amplitude of the detection mode angular displacement can be expressed as:

(13) $${\hat{\theta }}_{d}=\sqrt{A^{2}+B^{2}}=\frac{2\;L_{x}L_{y}\omega_{a}}{J_{d}}\;\frac{Q_{d}}{\sqrt{{\left(\omega_{d}^{2}-\omega_{a}^{2}\right)}^{2}Q_{d}^{2}+\omega_{d}^{2}\omega_{a}^{2}}}\Phi_{m}\alpha \;$$

The amplitude ${\hat{z}}_{d}$ of the displacement of the tube in the Coriolis mode, as indicated in Figure 1b, can now be expressed as:

(14) $${\hat{z}}_{d}=L_{y}\cdot {\hat{\theta }}_{d}$$

This displacement is measured, and it shows a linear relation with both the mass flow rate $\Phi_{m}$ and the actuation amplitude α. Therefore, in Section 4, we will plot the ratio ${\hat{z}}_{d}/a$, which is proportional to the mass flow and independent of actuation amplitude.

### 2.2. Sensor Design

According to Equations (13) and (14), to maximize the Coriolis mode displacement for a given mass flow rate $\Phi_{m}$ and actuation amplitude α, there are many parameters that need to be considered. Increasing the dimensions $L_{x}$ and $L_{y}$ will increase the sensitivity, but also lower the resonance frequencies $\omega_{a}$ and $\omega_{d}$, and increase the pressure drop over the sensor. Choosing $L_{x}$ and $L_{y}$ that are too large may even cause bending of the tube during the fabrication and curing of the device. Reducing the wall thickness will increase the sensitivity, but the minimum thickness is limited by the 3D-printing process. We have chosen a wall thickness of 230 µm, which could still be printed reliably without introducing leakage. With this wall thickness, choosing the inner diameter of 900 µm and the dimensions $L_{x}=14$ mm and $L_{y}=14$ mm will result in resonance frequencies around 2 kHz for the actuation mode and 1 kHz for the Coriolis mode, while the overall tube length of 42 mm still results in a very low pressure drop.

### 2.3. Measuring the Motion of the Tube with a Laser Doppler Vibrometer (LDV)

**Figure 2:**
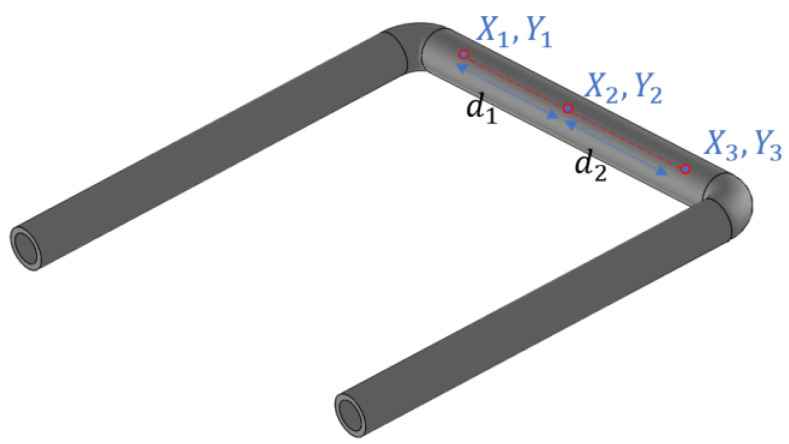
A U-shaped tube with three measurement points. There is no need for the second point $\left(X_{2},Y_{2}\right)$ to be in the exact center of the tube.

In the device presented in this paper, to measure the motion of the tube in swing mode, a laser Doppler vibrometer is chosen as a readout. Because of the round outer surface of the tube, measuring the displacement ${\hat{z}}_{d}$ in the Coriolis mode is challenging. Furthermore, the actuation axis can slightly shift due to a pressure gradient inside the tube. Therefore, we have chosen to measure the vibration of the tube in three points as indicated in Figure 2. Both the actuation amplitude α in (2) and the detection amplitude ${\hat{z}}_{d}$ from (14) are then calculated from the three measured amplitudes.

From the positions of the three points, we can calculate the distances between $d_{1}$ and $d_{2}$:

(15) $$d_{1}=\sqrt{{\left(X_{1}-X_{2}\right)}^{2}+{\left(Y_{1}-Y_{2}\right)}^{2}}$$

(16) $$d_{2}=\sqrt{{\left(X_{2}-X_{3}\right)}^{2}+{\left(Y_{2}-Y_{3}\right)}^{2}}$$

Taking the middle point as a reference, we can consider this as a one-dimensional problem along the x-axis with the middle point at the position $x_{2}=0$, point 1 at position $x_{1}=-d_{1}$ and point 3 at position $x_{3}=d_{2}$.

The measured displacement $z_{i}$ of each of the three points can be expressed in terms of an amplitude $a_{i}$ and phase $\varphi_{i}$:

(17) $$z_{1}\left(t\right)=a_{1}\sin\left(\omega_{a}t+\varphi_{1}\right)$$

(18) $$z_{2}\left(t\right)=a_{2}\sin\left(\omega_{a}t+\varphi_{2}\right)$$

(19) $$z_{3}\left(t\right)=a_{3}\sin\left(\omega_{a}t+\varphi_{3}\right)$$

In general, to find a least-squares fit of a line z=ax+b through three points $\left(x_{1},z_{1}\right)$, $\left(x_{2},z_{2}\right)$, $\left(x_{3},z_{3}\right)$, we can sum the values of $x,\;z,\;x^{2}$, and xz [24]:

(20) $$S_{x}=x_{1}+x_{2}+x_{3}=d_{2}-d_{1}$$

(21) $$S_{z}=z_{1}+z_{2}+z_{3}$$

(22) $$S_{x^{2}}=x_{1}{}^{2}+x_{2}{}^{2}+x_{3}{}^{2}=d_{1}^{2}+d_{2}^{2}$$

(23) $$S_{xz}=x_{1}z_{1}+x_{2}z_{2}+x_{3}z_{3}$$

to find the slope a and offset b:

(24) $$a=\frac{3S_{xz}-S_{x}S_{z}}{3S_{x^{2}}-S_{x}S_{x}}$$

(25) $$b=\frac{S_{z}-aS_{x}}{3}$$

By substituting (15) to (19) into (20) to (23), summing all time-dependent terms, which are sine waves with the same frequency but different amplitude and phase, and inserting the result in (24) and (25), we find the slope a and offset b as a function of time:

(26) $$a=A\sin\left(\omega t+\varphi_{A}\right)$$

(27) $$b=B\sin\left(\omega t+\varphi_{B}\right)$$

where a corresponds to the actuation angle $\theta_{a}$ of the tube (see Figure 1a), and b corresponds to the swing motion. The constants A, B, $\varphi_{A}$, and $\varphi_{B}$ depend on the measured amplitudes $a_{1}$, $a_{2}$, $a_{3}$ and phases $\varphi_{1}$, $\varphi_{2}$, $\varphi_{3}$ of the three points on the tube. The swing motion will be a combination of the motion due to Coriolis forces, $z_{d}$ (see Figure 1b), and motion due to the fact that the three measurement points may not be located exactly symmetrically with respect to the twist axis. However, the motion due to Coriolis forces will be 90 degrees out of phase with the actuation motion. Therefore, the amplitude of the Coriolis motion ${\hat{z}}_{d}$ as given by (14) is obtained by evaluating b(t) at the zero crossings of a(t):

(28) $${\hat{z}}_{d,\;measured}=B\sin\left(\varphi_{B}-\varphi_{A}\right)$$

In Section 4, we will plot the measured ratio ${\hat{z}}_{d}/\alpha$, which is given by:

(29) $${\left(\frac{{\hat{z}}_{d}}{\alpha }\right)}_{measured}=\frac{B\sin\left(\varphi_{B}-\varphi_{A}\right)}{A}$$

and compare that with the theoretical response derived in Section 2.1.

## 3. Fabrication Process

**Figure 3:**
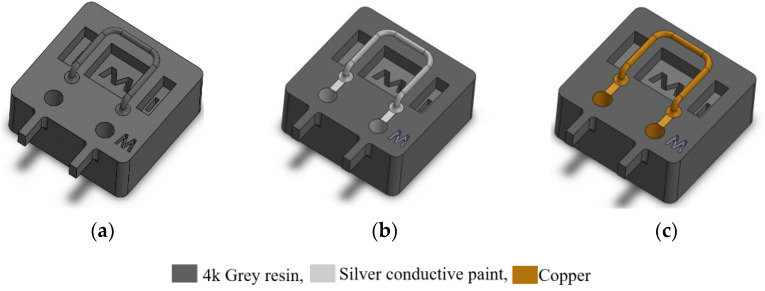
Schematic representation of a top view of the fabrication process. (**a**) Printing the whole structure, (**b**) applying silver conductive paint on the tube to provide a conductive track for applying the actuation current, and (**c**) copper plating on the surface of the tube to reduce the resistance of the conductive track.

**Figure 4:**
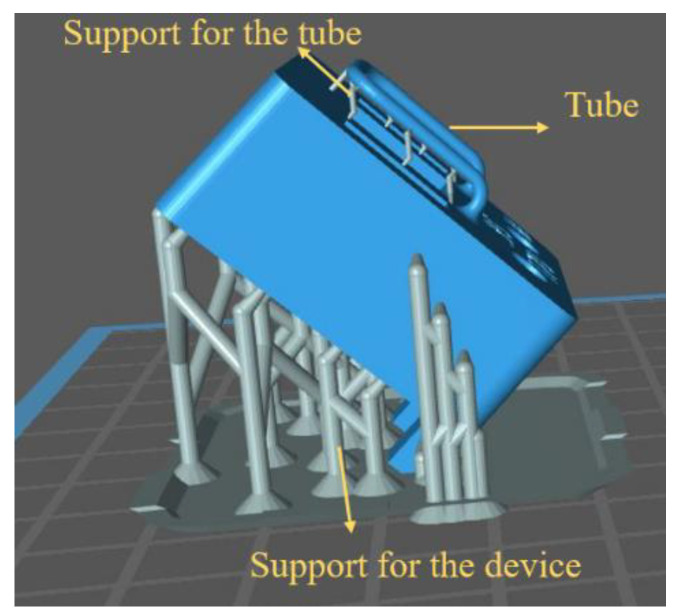
Adjusting the printed device angle to 45 degrees for high-quality printing on the Mini 4K Phrozen printer. Small support structures were added underneath the tube to prevent it from floating inside the resin during printing.

**Figure 5:**
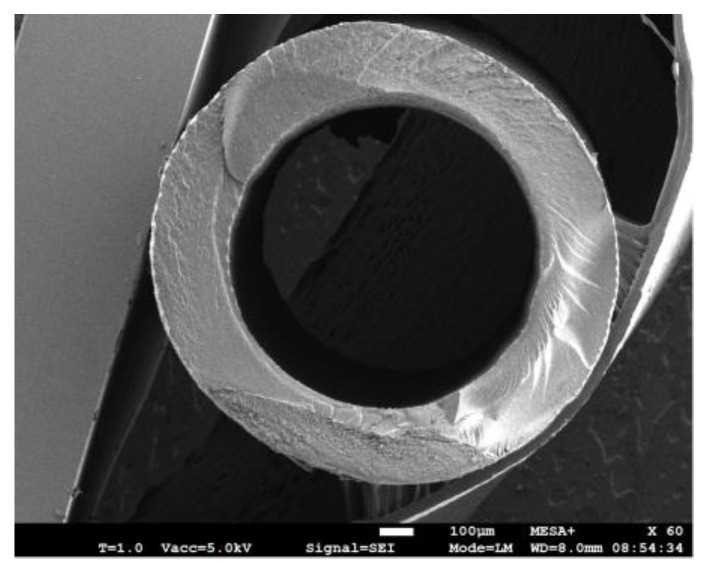
SEM photographs of the tube with a diameter of 900 µm, and a wall thickness of 230 µm. Based on the resolution of the printer that we used, this is the thinnest wall that could be reached for this U-shaped tube. A thinner wall caused leakage and a thicker wall decreased the sensitivity of the sensor due to the increased mass of the tube.

**Figure 6:**
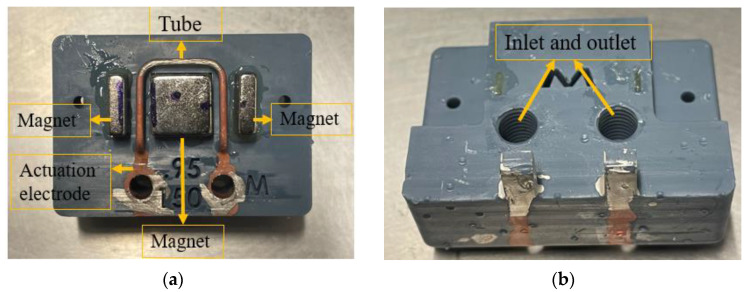
Photographs of the 3D-printed Coriolis mass flow sensor: (**a**) top view and (**b**) bottom view.

Figure 3 illustrates the fabrication process of the device. First, the whole mechanical structure of the device is 3D printed using a Phrozen Sonic mini 4K LCD 3D printer. The resin is Phrozen aqua grey 8k [25]. The entire structure is printed in 3 h and requires 12 mL of resin. Table 1 shows the properties of the 8k grey resin. This step, along with a post-printing process, includes cleaning and post-curing the structure. Then, the structure is immersed in a beaker of IPA in an ultrasound bath for 3 min to remove the uncured resin. In addition, a syringe is used to flush the IPA and uncured resin inside the tube to fill out the tube and is then placed in an ultrasonic cleaner for three more minutes to ensure that there is no uncured resin inside the structure. Then, the structure is dried with a nitrogen flow and UV-cured for one hour. Table 2 shows the detailed settings used for printing the design shown in Figure 3 with 8k grey resin. In addition, Figure 4 shows the 45-degree angle of the printed device on the printer stage for high-quality printing. This figure also shows the heavy supports on the bottom of the structure and much smaller supports underneath the tube to prevent the tube from floating inside the resin during printing. Figure 5 shows an SEM picture of a tube cross-section with a diameter of 900 µm and a wall thickness of 230 µm. In the second step, see Figure 3b, a thin layer of silver was applied to the outer surface of the tube to provide a conductive track on the tube to generate Lorentz force. The silver layer resulted in an electrical resistance of 9.8 Ω. A thin layer of copper was deposited by the electroplating, see Figure 3c [26], to reduce the resistance to 0.5 Ω. Figure 6 shows photographs of the completely assembled Coriolis mass flow sensor. The sensor contains a freely suspended U-shaped tube with a total length of 42 mm and a total mass of 0.05 g, a magnets holder, and a flow inlet and outlet.

## 4. Experimental Results and Discussion

**Figure 7:**
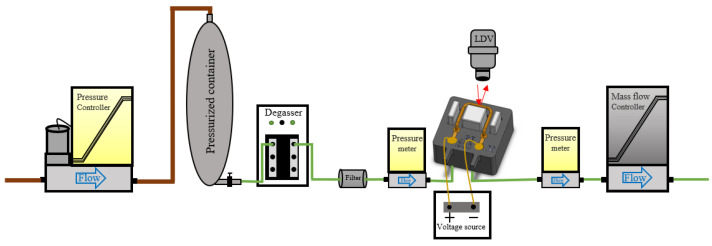
Block schematic of the experimental setup.

A block schematic of the experimental setup for applying liquid flows is shown in Figure 7. In this setup, a pressure controller sets the input pressure of a pressurized container that is filled with liquid, such as water or IPA. To prevent gas bubbles inside the liquid, a degasser is connected to the fluid container. A filter is inserted after the degasser to eliminate particles. Two pressure sensors are connected close to the inlet and outlet of the sensor that is being tested for measuring the pressure drop along the tube. A voltage source is used to apply the actuation signal to generate Lorentz forces. The displacement of the tube in both actuation and detection mode was detected by a laser Doppler vibrometer (LDV), a Polytec MSA-600 microsystem analyzer, as described in Section 2.3. A Bronkhorst mass flow controller is used as the last component in the line to control the mass flow rate. When testing with gases, the pressurized container and degasser are not needed and are removed from the setup. When measuring with liquids, the input pressure was between 4.5 and 5 bar. When measuring with gases, an input pressure of 8 bar was used.

**Figure 8:**
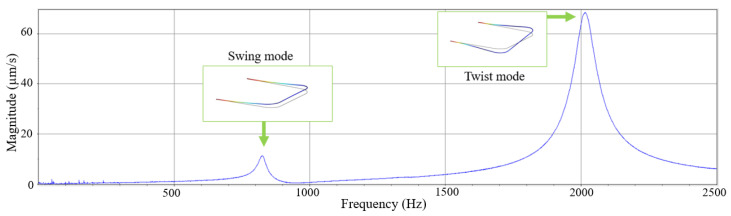
Measured vibration spectrum of air-filled tube detected by LDV. A periodic chirp signal with an amplitude of 30 mV was applied to the tube as an input voltage. The measured resonance frequencies and quality factors are 822 Hz and 27 for the swing mode, and 2015 Hz and 26 for the twist mode, respectively.

The measured vibration spectrum of the tube when filled with air at atmospheric pressure and with zero flow rate is shown in Figure 8. A periodic chirp with an amplitude of 30 mV was used as actuation voltage. Table 3 shows the measured resonance frequencies in both swing (detection) and twist (actuation) modes for water, IPA, and nitrogen with zero flow rate.

**Figure 9:**
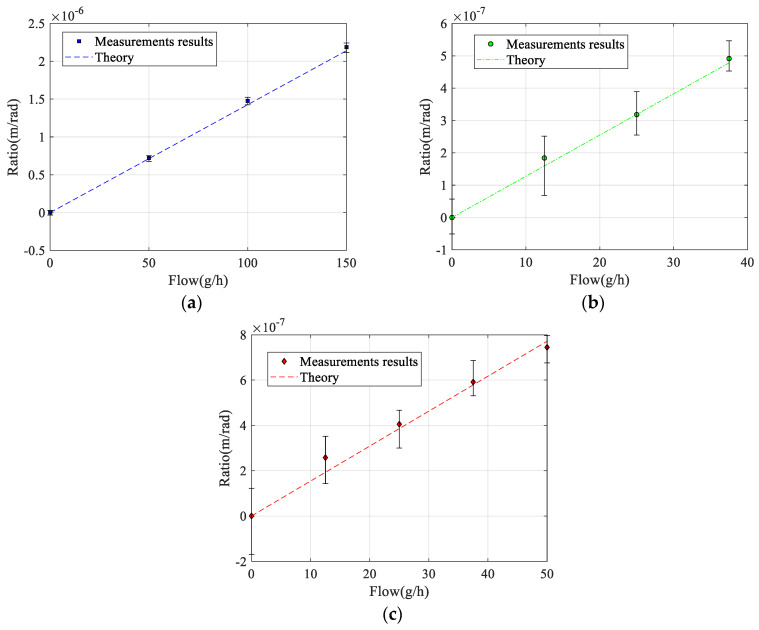
Measured and theoretical ratio between the Coriolis motion and the actuation amplitude according to Equations (29) and (14), respectively, as a function of mass flow of (**a**) water, (**b**) IPA, and (**c**) nitrogen. For all fluids, the same correction factor of 0.6 was applied to the model (14) such that the theoretical response matches the measurement results.

Figure 9 shows the measured ratio between the Coriolis motion and the actuation motion as a function of mass flow of water, IPA, and nitrogen. The device was actuated using a sinusoidal voltage with an amplitude of 40 mV at the twist mode resonance frequency given in Table 3, resulting in a twist mode vibration amplitude α between 1 and 1.5 mrad. Each point is the average of six measurements. The error bars correspond to the minimum and maximum measured values. For each measurement, the mass flow was first allowed to stabilize for at least 5 min, and then the ratio between Coriolis motion and actuation motion was calculated from the amplitude and phase of the three measurement points according to Equation (29). Figure 9 also shows the theoretical response calculated using the simple lumped element model presented in Section 2.1 and using the measured resonance frequencies listed in Table 3. The simple model overestimates the sensitivity by approximately a factor of 1.7 for all fluids. Therefore, in Figure 9, a constant correction factor of 0.6 was applied for all fluids such that the model matches the measured response. With this factor, the model correctly predicts the differences in sensitivity between the three fluids that result from the different resonance frequencies [27].

**Figure 10:**
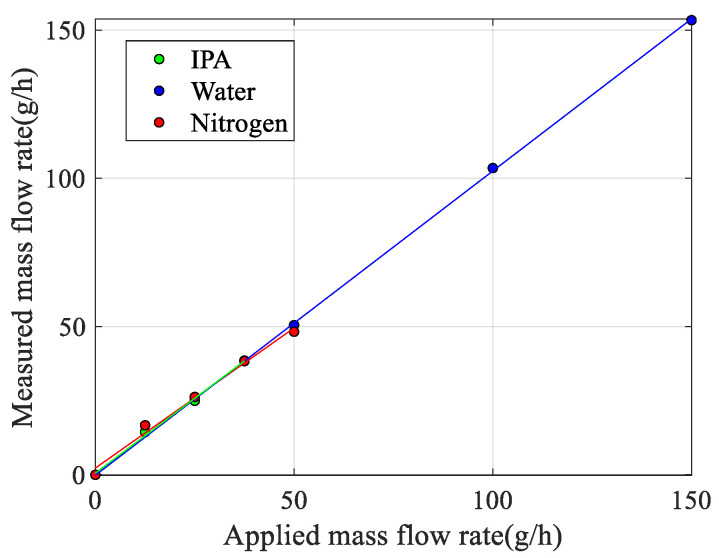
Measured mass flow rate as a function of applied mass flow rate for water, IPA, and nitrogen, using the theoretical response (14) to convert the measured response into the corresponding mass flow.

Apart from the correction factor of 0.6, the measured and theoretical responses match very well with each other. The fact that the model overestimates the sensitivity may have several reasons. The model is a simple lumped element approximation that assumes that the tube structures rotate around the twist and swing mode axes and ignores bending of the tube. Furthermore, the effect of the smooth bends in the actual device with a relatively large radius of curvature is not taken into account. Because of these bends, the Coriolis forces will result in a significantly smaller detection torque (4) because only the straight part of the tube segment with length $L_{x}$ is at a distance $L_{y}$ from the swing mode axis. Figure 10 shows all measured responses together when using the theoretical response (14) to convert the measured displacement amplitude into the corresponding mass flow. The slopes of the linear fit for water, IPA, and nitrogen are 1.03, 1.01, and 0.95, respectively. Ideally, these slopes should all be equal to 1.0. The difference in slopes is slightly more than the 2.6% found in [27] and could be due to a slight change in the vibration mode due to the pressure gradient introduced by the mass flow, which depends on the type of fluid and is not included in the model.

**Figure 11:**
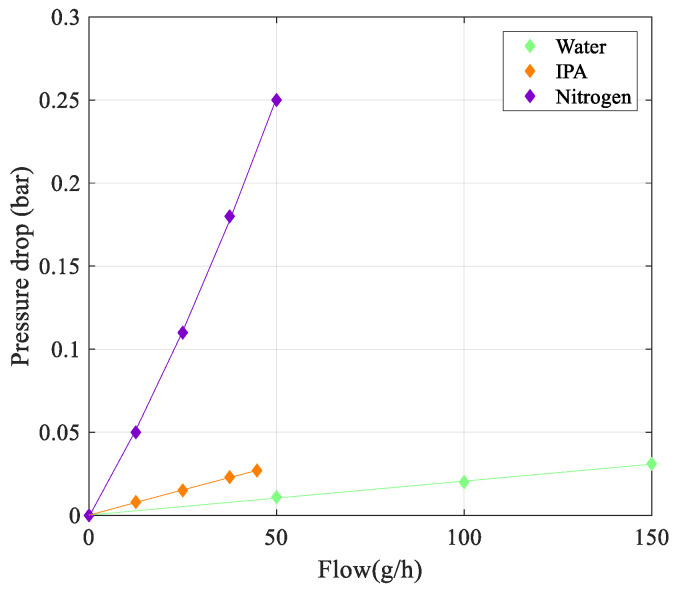
Measured pressure drop over the tube as a function of mass flow rate.

The pressure drop over the tube vs. flow rate is shown in Figure 11. An estimation of the pressure drop based on the Hagen–Poiseuille equation shows that the maximum pressure drop for water and IPA should be 1.1 mbar and 0.8 mbar, respectively [17]. The pressure drop for nitrogen is expected to be around 5 mbar, mainly due to the two 90-degree bends in the tube. As is clear from Figure 11, the measured pressure drop is significantly higher because of the extra pressure drop in the relatively long connecting tubing between the sensor and the two pressure sensors.

## 5. Conclusions

This paper reported the modeling, fabrication, and testing of a fully 3D-printed Coriolis mass flow sensor using 8k grey resin. The sensor consists of a U-shaped tube with a total length of 42 mm. The tube has a circular cross-section with an inner diameter of about 900 µm, and a wall thickness of approximately 230 µm. The sensor was actuated by Lorentz force and read out by a laser Doppler vibrometer. The measurement results showed a linear response for different flow rates. It results in a circular cross-section tube which decreases pressure drop over the tube during measurements. Future work will focus on reducing the wall thickness to decrease the tube mass and integrating the readout circuit on the sensor to eliminate the need for the laser Doppler vibrometer. Furthermore, additional research is needed to obtain the same sensitivity to mass flow for all fluids.

## Figures and Tables

**Table 1:** Properties of the 8k grey resin [25].

Specs	
Density	1.1 g/cm^3^
Surface hardness	85 shore D
Tensile modulus	2256 MPa
Flexural strength	54 MPa
Flexural modulus	1551 MPa

**Table 2:** Detailed setting for printing the structure with 8k grey resin by Mini 4K Phrozen printer.

3D Printer Setting Parameter	
Layer height	0.035 mm
Bottom layer count	6
Transition layer count	6
Exposure time	2.5 s
Light-off delay	13 s
Bottom exposure time	35 s
Lifting distance	6 mm
Lifting speed	60 mm/min
Retract speed	150 mm/min

**Table 3:** Measured resonance frequencies in twist and swing mode, and the quality factor in swing mode. During flow measurements, the device is actuated in resonance in the twist mode. Coriolis forces due to mass flow will actuate the swing mode at the vibration frequency of the twist mode.

Fluid	Resonance Frequency in Swing Mode (Hz)	Quality Factor in Swing Mode	Resonance Frequency in Twist Mode (Hz)
Water	611	18	1465
IPA	722	35	1756
Nitrogen	843	31	2056

## Data Availability

Not applicable.
